# Focal macromolecule delivery in neuronal tissue using simultaneous pressure ejection and local electroporation

**DOI:** 10.1016/j.jneumeth.2008.10.021

**Published:** 2009-03-15

**Authors:** Matthew Barker, Brian Billups, Martine Hamann

**Affiliations:** a Department of Cell Physiology and Pharmacology, Leicester University, Medical Sciences Building, P.O. Box 138, University Road, Leicester LE1 9HN, UK; b Department of Pharmacology, University of Cambridge, Tennis Court Road, Cambridge CB2 1PD, UK

**Keywords:** Electroporation, Pressure, Axonal tracing, Propidium iodide, Dextran amine, Transfection efficiency, HEK293, Auditory brainstem, Slice, Cerebellum, Hippocampus

## Abstract

Electroporation creates transient pores in the plasma membrane to introduce macromolecules within a cell or cell population. Generally, electrical pulses are delivered between two electrodes separated from each other, making electroporation less likely to be localised. We have developed a new device combining local pressure ejection with local electroporation through a double-barrelled glass micropipette to transfer impermeable macromolecules in brain slices or in cultured HEK293 cells. The design achieves better targeting of the site of pressure ejection with that of electroporation. With this technique, we have been able to limit the delivery of propidium iodide or dextran amine within areas of 100–200 μm diameter. We confirm that local electroporation is transient and show that when combined with pressure ejection, it allows local transfection of EGFP plasmids within HEK293 cells or within cerebellar and hippocampal slice cultures. We further show that local electroporation is less damaging when compared to global electroporation using two separate electrodes. Focal delivery of dextran amine dyes within trapezoid body fibres allowed tracing axonal tracts within brainstem slices, enabling the study of identified calyx of Held presynaptic terminals in living brain tissue. This labelling method can be used to target small nuclei in neuronal tissue and is generally applicable to the study of functional synaptic connectivity, or live axonal tracing in a variety of brain areas.

## 1 Introduction

Electroporation uses electrical pulses to create transient pores in the plasma membrane thereby allowing macromolecules to be introduced within cells. Electroporation is an efficient way to introduce genes as an alternative to viral vectors (Gehl, 2003; Golzio et al., 2004) which present potential problems like toxicity, immune and inflammatory responses or to the gene gun delivery approach (O’Brien and Lummis, 2004) which can cause tissue damage. The spectrum of biological preparations to which electroporation has been applied includes single cells (Olofsson et al., 2003), cell suspensions (Pavlin et al., 2007), adherent cultured cells (Teruel et al., 1999), whole cerebellum (Yang et al., 2004) or brain slice preparations (Murphy and Messer, 2001), whole chick or mouse embryos (Inoue and Krumlauf, 2001; Krull, 2004) and whole tissues such as muscle or lung (Aihara and Miyazaki, 1998; Dean et al., 2003). Electroporation usually targets a large population of cells and in most cases, electric pulses are delivered between two plate electrodes in a solution containing cells (Teruel et al., 1999; Pavlin et al., 2007) or by inserting a pair of electrodes into the tissue (Aihara and Miyazaki, 1998; Osumi and Inoue, 2001; Dean et al., 2003) or across a slice (Murphy and Messer, 2001). Whereas the electrical environment within the chamber is described by the field strength which depends upon the voltage and the distance between the electrodes, it has been shown the membrane surface facing the anode develops pores of larger radius and lower density (Tekle et al., 1994). While pores of adequate diameters will allow exogenous material to diffuse into the cytoplasm, large or numerous pores might cause chemical imbalances leading to eventual cell death (Weaver, 1993). Spatial resolution is therefore necessary for efficient and homogeneous macromolecule delivery and maintenance of cell viability. Electroporation is successful for transferring DNA and macromolecules locally to individual cells in intact tissue (Haas et al., 2001; Bestman et al., 2006). Instrumentation has also been developed to achieve electroporation of a small number of cells in suspension (Tsong and Kinosita, 1985; Marszalek et al., 1997) and of a small number of adherent cells grown on a substratum (Zheng and Chang, 1991; Teruel and Meyer, 1997; De Vuyst et al., 2008). These electroporation devices create homogeneous electric fields across fixed distances of 0.1–5 mm, several times larger than the size of a single mammalian cell. But localised electroporation has yet to be performed when dealing with slices. A double-barrelled electrode has been used to perform localised electroporation and target gene expression in avian embryo (Atkins et al., 2000). This method required pools of DNA injected in the embryo as well as suction of the tissue within the electrode. Tjelle et al. (2006) developed a device combining ejection and electroporation to inject DNA into muscles of large animals and humans. The DNA was injected via two needles inserted in the muscles and the needles also served as electrodes allowing focal ejection of the DNA at the site of electroporation. Here we present data obtained using a similar design that combines ejection and electroporation through a double-barrelled glass micropipette to precisely inject impermeable macromolecules to target small nuclei in neuronal tissue. We show that (1) the double-barrelled micropipette design allows us to reduce the distance between the electrodes therefore achieving focal electroporation and dye delivery (2) the higher spatial resolution of focal electroporation increases cellular viability compared to when electroporation is performed between two electrodes separated from each other; (3) efficient and localised delivery of propidium iodide or dextran amine in cell culture or in slices are achieved within areas of 100–200 μm diameter; (4) this method can be used to label axon pathways to probe synaptic connections in living tissue.

## 2 Materials and methods

### 2.1 Pressure ejection

**Fig. 1:**
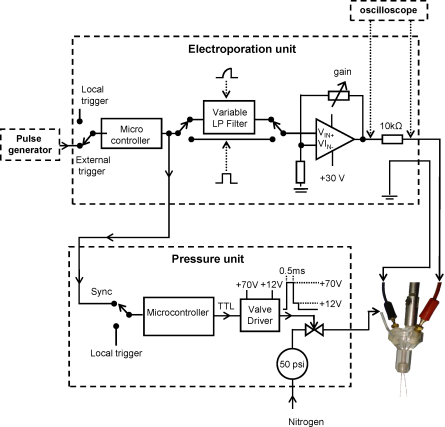
Circuit diagram of the electroporation and pressure ejection apparatus. The device consists of a pressure unit coupled to an electroporation unit. In both units, microcontrollers allow the number of pulses, the pulse width and the period between pulses to be set. The electroporation unit can be triggered manually (local trigger) or via a pulse generator (external trigger). The output of the operational amplifier (OPA 548) delivers up to 30 V depending on the gain setting. A 10 kΩ resistance is coupled to an oscilloscope to measure the resistance during the electroporation (see Section 2.2). A variable low pass (LP) filter allows adjustment of the rise time of the pulses and this can be bypassed to apply square pulses. The pressure unit can be either triggered manually or synchronised with the electroporation unit. The pressure unit is connected to a pressure source of 50 psi with attendant regulators and valves. An inactive 3-way solenoid valve connects the micropipette to atmospheric pressure. When activated, the valve permits the application of a positive pressure pulse of specified duration and pressure to the micropipette. The solenoid valve driver applies 70 V for 0.5 ms and 12 V for the remainder of the pulse time (between 0.5 and 900 ms). Dotted lines around the units indicate that units were contained in separate boxes. A suitable micropipette holder allows electroporation via the two silver chloride wires which are inserted in the double-barrelled electrode and pressure is delivered to both barrels via the polythene catheter.

**Fig. 2:**
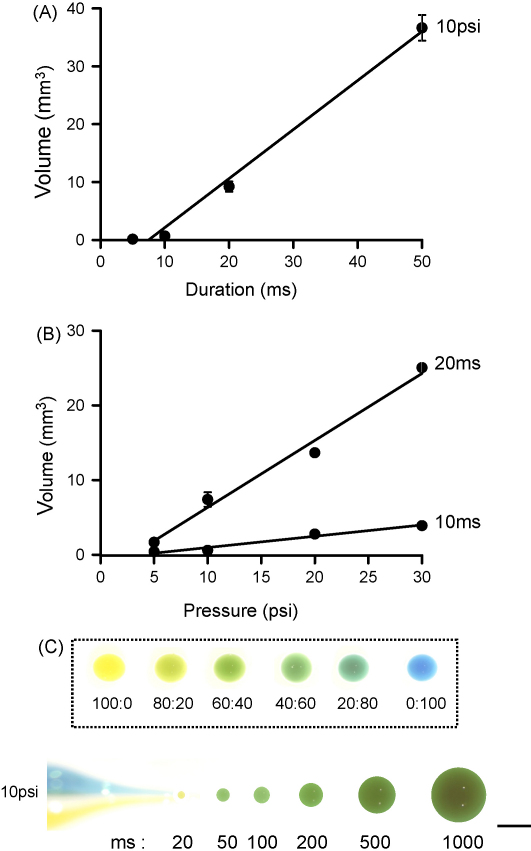
Calibration of the pressure unit. (A) Relationship between the volume of cresyl violet ejected and the ejection duration, for a constant pressure of 10 psi. (B) Relationship between the volume of cresyl violet ejected and the pressure for a constant ejection duration of 10 ms (bottom) or 20 ms (top). Relationships were fitted with linear regressions with *r*^2^ = 0.99 (A), *r*^2^ = 0.99 (B, bottom) and 0.93 (B, top). (C) Top. Calibration chart showing 100 μl spheres containing blue and yellow dyes previously mixed with a different proportion. Bottom. Double-barrelled pipette with blue and yellow dyes in the separate barrels. The droplets shown on the right were ejected at 10 psi and with increasing ejection durations. Ejected droplets were green indicating that the blue and yellow dyes are mixed together with a similar ratio (there is a slight bias towards the yellow ejected dye at the lowest duration of 20 ms). Scale bar is 10 mm. (For interpretation of the references to colour in this figure legend, the reader is referred to the web version of the article.)

The pressure unit (Fig. 1) is connected to a pressure source of nitrogen equipped with a two stage regulator, providing an initial delivery pressure of 50 psi. Pressure is applied through a pipette (see below) and a three-way solenoid valve (catalogue No 9-284-900, General Valve Corp. NJ, USA) connects the pipette to the atmosphere. When activated, the solenoid valve permits application of brief positive pressure pulses of specified duration (from 1 to 100 ms) and pressure (up to 50 psi) to the pipette. The pressure unit can be triggered manually or synchronised with the electroporation unit. Pulses of adjustable duration are applied via a solenoid driver circuit (valve driver) which initially applies 70 V for 0.5 ms in order to power the valve. The valve is then held on by 12 V for the remainder of the pulse. A microcontroller (catalogue reference number PIC 18F242, Microchip Ltd., Wokingham, UK) sets the number of pulses, the pulse width and the period between pulses although single pulses were generally delivered. We calibrated the pressure ejection delivery system by ejecting 1% cresyl violet dissolved in water into an oil interface. This allowed us determining the ejection pressure and duration parameters that will be subsequently used in cell culture or in slices (Fig. 2). Fig. 2A shows a linear relationship between the duration varying from 5 to 50 ms and the ejected volume of cresyl violet for a pressure of 10 psi. Fig. 2B shows a linear relationship between the pressure varying between 5 and 30 psi and the ejected volume of cresyl violet. As we delivered dyes within cell cultures or slices we used the minimal pressure and duration that were linearly related to the volume, i.e. a pressure of 10–20 psi for 10–20 ms. We tested the possibility of using the two barrels separately to contain different solutions. When the double-barrelled pipette is filled with blue and yellow dyes in separate barrels the presence of green droplets (indicating that dyes are mixed together at a similar ratio) could be achieved for pulse durations exceeding 20 ms (Fig. 2C). In our experiments each pipette barrel was filled with the same medium to a quarter of its length.

### 2.2 Electroporation

Electroporation is achieved by applying 30 V square pulses through a TGP110 pulse generator (Thurlby Thandar Instruments, Huntingdon, UK, Fig. 1). A microcontroller (PIC 18F242, Microchip Ltd.) sets the number of pulses, the pulse width and the period between pulses. Pulses were sent to an operational amplifier (OPA 548, Texas Instruments Inc., Dallas, Texas, USA) via a variable low pass filter (LT1062, Linear Technology, Buckinghamshire, UK) that allows pulse shaping. The present study combines electroporation with pressure ejection and used 5 ms square wave pulses applied at 100 Hz for 2–10 s with pressure ejection occurring mid-way through the electroporation. The resistance of the circuit (comprising the pipette, the media and the tissue) was deduced from voltage measurement across a 10 kΩ series resistance. Thus it is possible to monitor whether the pipette was clogged or broken during the electroporation process. For a 30 V pulse, a typical voltage measured across the 10 kΩ series resistance indicated values of 0.1 mA and 290 kΩ across the circuit. During voltage stimulation, the electroporation field will depend on the electrode resistance. Similar results were obtained whether using a voltage stimulator as described above or injecting 0.1 mA across a current stimulator (modified DS2A stimulator, Digitimer Ltd., Welwyn Garden City, UK). The monitoring connection could also introduce an unwanted connection to ground that could shunt the electroporation electrode. Similar results were obtained when using an un-earthed Tektronix THS710A oscilloscope (Bracknell, Berkshire, UK).

### 2.3 Pipette

Ejection and electroporation were performed with double-barrelled borosilicate capillary glass electrodes with an outer diameter of 2 mm, a wall thickness of 0.30 mm and a septum of 0.22 mm (Harvard Apparatus Ltd., Edenbridge, UK). The pipette was pulled in a vertical PE-21 puller (Narishige Scientific Instruments, Tokyo, Japan) and the electrode diameter for both tips were 142 ± 29 μm (*n* = 8). The pipette was held in a custom-made pipette holder (Fig. 1) with threaded end-pieces and O-rings to form a leak-proof seal around the pipette. This holder permits substance ejection via pressure to be combined with electroporation via the two pipette barrels. The holder is connected to an electroporation unit via two silver chloride wires (one per pipette barrel) and to the pressure unit via a polyethylene catheter linked to an electromechanical valving system.

### 2.4 Ejection combined to whole tissue electroporation

Ejection combined to whole tissue electroporation was performed with the same pipette described above except that in this case one barrel of the pipette served as the cathode and a bath electrode served as the anode. The anode consisted of a flattened piece of platinum onto which the tissue of interest was glued. Ejection and electroporation occurred in a similar way as previously described.

### 2.5 Dissection and slicing

Twenty Lister Hooded rats, 12–21-day-old were culled in accordance with the United Kingdom Home Office regulations. The brain was transferred immediately to ice-cold low sodium artificial solution containing (in mM): 250 sucrose, 2.5 KCl, 10 glucose, 1.25 NaH_2_PO_4_, 26 NaHCO_3_, 0.5 ascorbic acid, 0.1 CaCl_2_, and 4 MgCl_2_ (pH 7.4 when gassed with 95% O_2_: 5% CO_2_). Brainstems or cerebella were dissected out and glued onto a mounting plate placed within a plastic dissection chamber before being sectioned on a VT1000S vibroslicer (Leica Microsystems Ltd., Milton Keynes, UK) to the appropriate depth where the electroporation and dye ejection were performed. Extracellular medium was then changed to an artificial cerebrospinal (aCSF) solution containing (in mM) 125 NaCl, 2.5 KCl, 10 glucose, 1.25 NaH_2_PO_4_, 26 NaHCO_3_, 2 sodium pyruvate, 3 myo-inositol, 0.5 ascorbic acid, 2 CaCl_2_, and 1 MgCl_2_, pH 7.4 when gassed with 95% O_2_: 5% CO_2_. Dye ejection and electroporation were performed in the aCSF solution under binocular control using a MZ7.5 stereomicroscope (Leica Microsystems Ltd.). Brainstems or cerebella attached to their mounting plate were then transferred to an incubation chamber containing aCSF gassed with 95% O_2_:5% CO_2_ at 37 °C for a period of 3–4 h. Final slicing was performed by transferring the mounting plate containing the brainstem or the cerebellum into the vibroslicer dissection chamber containing ice-cold low sodium artificial solution. Hundred microns thick slices were cut and transferred to the stage of an Eclipse TE2000-U inverted microscope (Nikon Instruments Ltd., Kingston, UK) or a Fluoview IX70 confocal microscope (Olympus Ltd., Watford, UK) for final observation.

When electrophysiological recordings were performed in principal neurones of the medial nucleus of the trapezoid body, dextran amine dyes were delivered in the tissue as described previously and slicing was performed as described above, immediately after dye delivery. Slices were maintained in aCSF for 1 h at 37 °C and for another hour at room temperature before electrophysiological recording. This ensured that all labelled calyces could be traced back at least as far as the midline where the stimulating electrode was place. Therefore all labelled calyces could be stimulated.

### 2.6 HEK293 cell culture

HEK293 cells were maintained in minimal essential medium with Earle's Salts (with GlutaMAX™ I) supplemented with 10% foetal bovine serum, 1% non-essential amino acids, 1% penicillin/streptomycin and 1% fungizone (all components from Invitrogen Ltd., Paisley, UK) at 37 °C in a humidified atmosphere of 5% CO_2_ and 95% air. Cell cultures were split upon reaching 70–80% confluency and transferred to either culture flasks or 35 mm Petri dishes for experimentation. During experimentation, cells were perfused with a HEPES solution containing (in mM): 150 NaCl, 10 HEPES, 2.5 KCl, 11 glucose, 1 MgCl_2_ and 2.5 CaCl_2_ at pH 7.3 (HEPES buffered solution also used as described below).

### 2.7 Cerebellar and hippocampal slice cultures

Tissue preparation and electroporation were performed on 3–4-day-old Lister Hooded rats as described previously. The vermis of the cerebella and the hippocampus were sliced sagittally into 150 μm thick slices with the VT1000S vibroslicer (Leica Microsystems). The slices were cultured in Falcon 8.0 μm pore size cell culture inserts (Becton Dickinson Labware, Oxford, UK) containing 96% Neurobasal medium, 2% heat-inactivated horse serum, 1% B27 supplement, 0.5 mM l-glutamine, 1% penicillin–streptomycin (all from Invitrogen). Slices were maintained for 24–48 h in a humidified atmosphere at 37 °C and 5% CO_2_. Slices were finally fixed with 4% paraformaldyhyde before being mounted with Vectashield Hardset mounting medium (Peterborough, UK), covered and visualised with an Olympus IX70 confocal microscope (Olympus Ltd., Watford, UK).

### 2.8 Dyes and plasmids

We used local ejection combined with local electroporation to transfer impermeant macromolecules (i.e. propidium iodide, dextran amine, EGFP plasmid) within cells that were either in slices or in culture (Meldrum et al., 1999). Propidium iodide and Sytox green were also used to label necrotic cells and were then added to the perfusion medium (Meldrum et al., 1999; Evans and Cousin, 2007). Propidium iodide (Invitrogen) was diluted in the HEPES buffered solution previously described at a concentration of 1.5 mM for pipette application or bath applied at 1 μM. Fluorescent dextran tetramethylrhodamine (fluoro-ruby) 10,000 MW (Invitrogen) was made up as a 10% stock solution in 0.4 M KCl and further diluted at 1:10 in the HEPES solution described above before application via the pressure pipette. Sytox green (Invitrogen) was diluted in the HEPES buffered solution at 1 μM and bath applied to label necrotic cells (Evans and Cousin, 2007) immediately after electroporation without or with 0.1% Triton X-100 (Sigma). The GFP plasmid pEGFP-N1 (Clontech-Takara Bio Europe) was diluted in the HEPES buffered solution and applied at 1 μg μl^−1^ via the pressure pipette.

### 2.9 Electrophysiology

Transverse brainstem slices (200 μm thick) were cut as previously described (Barnes-Davies and Forsythe, 1995) in low artificial sodium solution before being transferred to the experimental chamber containing aCSF for recording. Medial nucleus of the trapezoid body fibre principal neurones and calyces of Held were visualized with infrared differential interference contrast (DIC) optics on an E600FN microscope (Nikon Instruments Ltd., Kingston, UK) with a 60× N.A. 1.0, water-immersion fluor lens. Cell attached and whole-cell patch-clamp recordings were made from the postsynaptic cell using thick-walled glass pipettes (GC150F-7.5, Clark Electromedical, Harvard Apparatus, Ltd. Kent, UK) with an Axopatch 200B amplifier (Molecular Devices, Sunnywale, CA, USA), filtered at 5 kHz (8-pole Bessel filter) and sampled at 20 kHz. Currents were recorded with pCLAMP 9 software (Molecular Devices). Whole-cell access resistances of less than 10 MΩ were compensated >70% with a 10 μs lag time. The intracellular solution contained (mM) 110 CsCl; 40 HEPES; 10 TEA-Cl; 12 Na_2_-phosphocreatine and 1 EGTA (pH adjusted to 7.3 with CsOH); 2 mM QX314 was added to the intracellular solution to block postsynaptic sodium currents and improve the quality of the voltage-clamp. Excitatory postsynaptic currents were elicited with a bipolar platinum electrode consisting of two platinum wires 300 μm apart placed above and below the slice at the midline (Fig. 8E). Electrical pulses of 2–8 V and 0.2 ms duration were provided by a Digitimer DS2 A isolated stimulator (Digitimer Ltd., Welwyn Garden City, UK) triggered by the pCLAMP software.

### 2.10 Analysis

#### 2.10.1 Profile areas

**Fig. 3:**
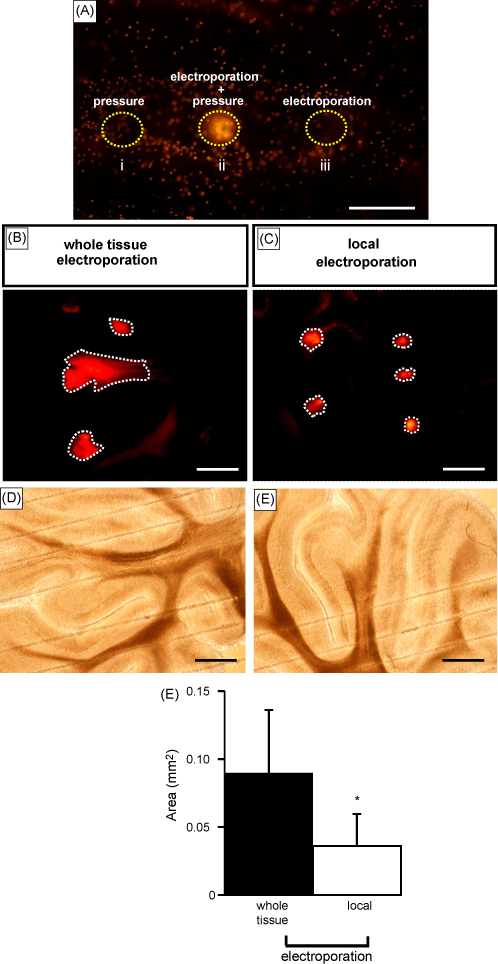
(A) Local electroporation and local ejection are required for propidium iodide delivery. (A) Propidium iodide was here delivered through a double-barrelled pipette within a brainstem slice, using 10 psi, 10 ms pressure alone (i), combined with local electroporation (ii) or using local electroporation solely (iii). (B–F) Labelled areas differ with the electroporation method. (B and C) Propidium iodide was delivered within the cerebellum using pressure ejection in conjunction to whole tissue electroporation via two separate electrodes (B) or in conjunction to local electroporation via a double-barrelled electrode (C). Pressure of 10 psi for 10 ms was applied through the same double-barrelled pipette in B and C but in B, the two electrodes consisted of one electrode in the pipette barrel and the other electrode in the bath whereas in C, the two electrodes were housed in isolated chambers of the same double-barrelled pipette. Both slices in B and C were taken at the level of propidium iodide ejection. Lines delimitate the sites of propidium iodide ejection and electroporation. D and E are the transmitted light images of B and C respectively. Horizontal lines are nylon strings used to maintain the slice on the bottom of the perfusion chamber. (F) Histogram summarising the labelled areas as in B and C, using two separate electrodes (black) or a double-barrelled electrode (white) respectively. Labelled areas while using local electroporation or whole tissue electroporation were 0.036 ± 0.023 mm^2^ (*n* = 16) and 0.089 ± 0.046 mm^2^ (*n* = 19) respectively. Scale bar is 100 μm in A and 500 μm in B–E. *Significant difference at *p* < 0.001.

Analysis of labelled profile areas (Fig. 3B and C) was performed on 3–5 ejection sites per cerebellar slice per rat and 5 rats were used per condition. Analysis was performed at the level of the ejection site using the Image-J 1.36 freehand selection tool allowing selecting the contour of the labelled area together with the Image-J 1.36 area calculator plugins allowing estimating the surface areas.

#### 2.10.2 Number of dead cells

Dead cells were labelled with bath applied 1 μM propidium iodide (Meldrum et al., 1999) subsequent to electroporation. Cells labelled with propidium iodide were counted in slices cut 600 μm below the level of ejection of a HEPES buffered solution. HEPES buffered solution ejection was combined to local electroporation or to whole tissue electroporation (see above). Analysis was performed in squares (100 μm × 100 μm) placed throughout the granule cell layer of the cerebellum (2–4 squares per slice). Three rats were used per condition.

### 2.11 Delivery and transfection efficiency within a localised area

Following electroporation, a circular area delimitating all propidium iodide labelled cells was applied. The diameter of the circle was determined by the outermost propidium iodide labelled cells and the delivery efficiency was calculated as the percentage of propidium iodide labelled cells within this area (Meldrum et al., 1999). As GFP could not be visualised at the time of electroporation, the transfection efficiency was calculated by the following method. Calculation of transfection efficiency was performed by analysing the number of propidium iodide positive cells that expressed also GFP at 24 h after electroporation then multiplying by the delivery efficiency.

### 2.12 Statistics

Data are expressed as the mean ± S.D. and statistical significance (*p* < 0.05) was tested with 2-tailed *t*-tests.

## 3 Results

### 3.1 Combining local ejection with local electroporation allows discrete labelling and reduction of cell death in slices

**Fig. 4:**
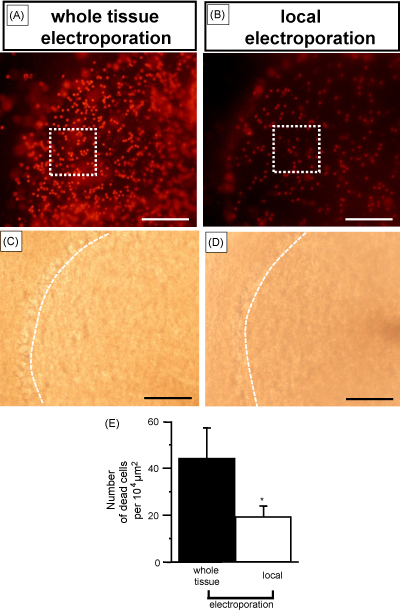
Cell death differs with the electroporation method. Dead cells were labelled with bath applied 1 μM propidium iodide following pressure ejection of HEPES solution (10 psi for 10 ms) combined to local or whole tissue electroporation. Ejection of HEPES solution and electroporation were performed in the whole cerebellum and slices were then cut and perfused with propidium iodide. Cell death was counted in slices cut 600 μm below the level of HEPES solution local ejection. (A) Electroporation was performed using either two separate electrodes (whole tissue) or (B) a double-barrelled electrode (local). Analysis was performed in squares (100 μm × 100 μm) placed throughout the granule cell layer of the cerebellum (2–4 squares per slice). The two squares in dotted lines represent examples of areas in which cell counting has been performed. For quantification all fluorescent cells that were in focus were included. C and D are the transmitted light images of A and B respectively. The dashed line delimitates the Purkinje cell layer in both C and D. (E) Histogram summarising the number of dead cells per 10,000 μm^2^ labelled with perfused propidium iodide after electroporating with two separate electrodes (black, see A) or with a double-barrelled electrode (white, see B). Data are from 63 and 66 squares (6 slices each) from A and B respectively. *Significant difference at *p* < 0.002. Scale bar is 100 μm in A–D.

We developed a method in which pressure ejection of dye (or plasmid) and electroporation occur at the level of a double-barrelled pipette tip (combined local ejection-electroporation method (Fig. 1). As a result of the design, pressure and electroporation occur at the level of the pipette tip. We applied propidium iodide directly in brainstem slices and found that neither pressure alone nor electroporation alone were able to label cells (Fig. 3Ai and iii respectively). Localised dye delivery was only achieved when localised pressure was combined to local electroporation via the double-barrelled pipette (shown by the higher density of labelling in Fig. 3Aii). We then delivered propidium iodide within the cerebellum using pressure ejection in conjunction to whole tissue electroporation via two electrodes or in conjunction to local electroporation via a double-barrelled electrode. Slices were subsequently cut and labelled areas using both methods are shown in Fig. 3B and C respectively. We found that labelled areas were smaller (of about 200 μm diameter) and less variable when using local electroporation (summarised in Fig. 3F). We also quantified necrotic cell death produced after combining ejection of HEPES buffered solution to either local electroporation or to whole tissue electroporation. HEPES buffered solution was first applied within whole cerebella using one of the electroporation methods mentioned above. Cerebellar slices were then cut 600 μm below the level of ejection of the HEPES buffered solution and perfusion of propidium iodide onto the slices allowed quantifying necrosis. The number of necrotic cells was quantified in relationship to the combined ejection and local electroporation method (Fig. 4B) or to the combined ejection and whole tissue electroporation (Fig. 4A). We found that the number of necrotic cells (labelled with propidium iodide) was significantly higher when using whole tissue compared to local electroporation (44 ± 13 per 10,000 μm^2^ compared to 19 ± 5 per 10,000 μm^2^ respectively, *p* < 0.002, summarised in Fig. 4E).

### 3.2 Electroporation is necessary for dye insertion and forms transient pores

**Fig. 5:**
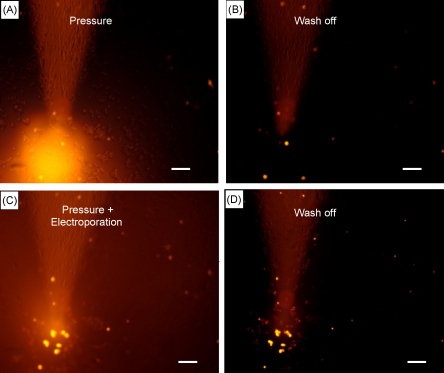
Electroporation is required to label cultured HEK293 cells. (A) Pressure ejection of propidium iodide (20 ms 15 psi) without electroporation using a double-barrelled pipette. (B) Subsequent wash off of the dye shows a few cells that have taken up the dye. (C) Pressure ejection of propidium iodide (20 ms, 15 psi) with electroporation using the same double-barrelled pipette (electrode). (D) Subsequent wash off of the dye shows the area of labelled cells that have inserted the dye. Scale bar is 100 μm from A to D.

**Fig. 6:**
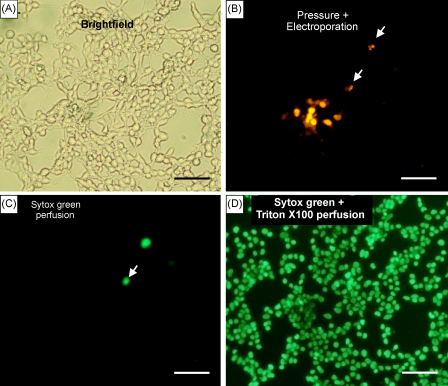
Electroporation induces transient pore formation in cultured HEK293 cells. (A) Brightfield image of cultured HEK293 cells. (B). Pressure ejection (10 psi, 10 ms) of propidium iodide combined to local electroporation using a double-barrelled electrode is followed by wash off and shows an area of labelled cells as well as one labelled cell remote from the ejection site shown by the arrow. (C) Subsequent bath application of Sytox green followed by wash off shows only one labelled cell that corresponds to the labelled cell indicated by the arrow in B. (D) Final bath application of Sytox green with Triton X-100 to permeabilise the membranes is followed by a wash off and shows that all cells have taken up Sytox green. Scale bar is 20 μm from A to D.

We used cultured HEK293 cells to quantify the delivery efficiency of propidium iodide. We previously showed propidium iodide was unable to enter cells within slices when applied either by pressure alone or by electroporation on its own. Similar results were obtained when applying propidium iodide on cultured HEK293 cells and Fig. 5C and D shows that propidium iodide was efficiently delivered in 66 ± 18% (*n* = 5) of HEK293 cells when cells were electroporated during pressure application. When propidium iodide was delivered by pressure alone (Fig. 5A and B) occasional labelling of one or two cells was likely to be due to labelling of necrotic cells. We checked whether the pores formed by electroporation were transient. If resealing takes place, then any subsequent permeability of the membrane can be attributed to the membrane becoming leaky during the necrotic process. We first applied propidium iodide locally via the combined ejection-local electroporation method and then perfused the cell culture with Sytox green (Fig. 6B and C) to check for necrosis (Evans and Cousin, 2007). We found that Sytox green did not penetrate cells containing propidium iodide thereby confirming that local electroporation formed transient pores that resealed after the electroporation process. As a control to assess permanent pore formation within all cells, we used the detergent Triton X-100 to permeabilise the cell membranes in combination with Sytox green. In this case we show that all cells were labelled with Sytox green (Fig. 6D) indicating that Triton X-100 triggered cell death.

### 3.3 The combined ejection and local electroporation method allows local plasmid transfection and EGFP expression

**Fig. 7:**
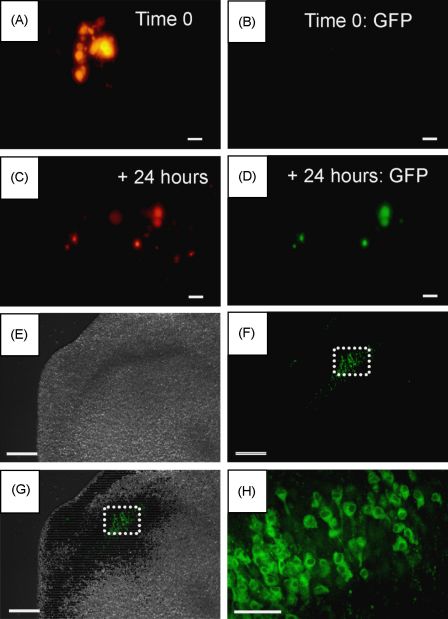
Cultured HEK293 cells express GFP following electroporation using a double-barrelled electrode. Propidium iodide and PEGFP-N1 GFP plasmid are simultaneously ejected (10 ms, 10 psi) via the double-barrelled pipette while electroporating via the two pipette barrels. (A) After wash off, HEK293 cells are labelled with propidium iodide. (B) Similar field of view as in A showing the absence of green labelled HEK293 cells immediately after plasmid ejection and wash off. (C) Same cultured cells as in A and B after 24 h showing propidium iodide labelled cells that have migrated from their original location in the culture dish. (D) Same field of view as in C showing that those cells are also expressing GFP 24 h after the plasmid ejection and integration. Scale bar is 20 μm from A to D.

The transient nature of the pore formation suggests that cells are viable immediately after electroporation (and dye ejection). We also tested whether HEK293 cells were able to express EGFP after transfecting with the pEGFP-N1 plasmid. We used the combined ejection and local electroporation method to deliver pEGFP-N1 to HEK293 cells. We included propidium iodide in the pipette as a means to trace which cells had been successfully electroporated (red cells) and which cells were subsequently able to express EGFP after 1 day in culture (green cells). Fig. 7 shows that HEK293 cells were initially labelled with propidium iodide solely (A and B). In those conditions the delivery efficiency was 66%. After 1 day in culture, propidium iodide labelled HEK293 cells also expressed EGFP as shown by the presence of green labelling. The transfection efficiency measured in this condition was 52 ± 10% (*n* = 6) indicating that (i) propidium iodide contained within cells still allowed the expression of the EGFP plasmid (ii) the combined ejection-local electroporation method can be used for transfecting plasmids in about half of the HEK293 cells. We next used cerebellar and hippocampal slice cultures to characterize local EGFP-mediated gene expression in cells within the intact tissue circuitry. Green fluorescence was observed after 24–48 h in culture. Labelled areas in cerebellar and hippocampal slices were 0.08 ± 0.03 mm^2^ (Fig. 7, 8 slices, 2 rats) and 0.07 ± 0.04 mm^2^ (6 slices, 2 rats) respectively, corresponding to diameters of around 350–500 μm.

### 3.4 The combined ejection and local electroporation method allows tracing presynaptic axons

**Fig. 8:**
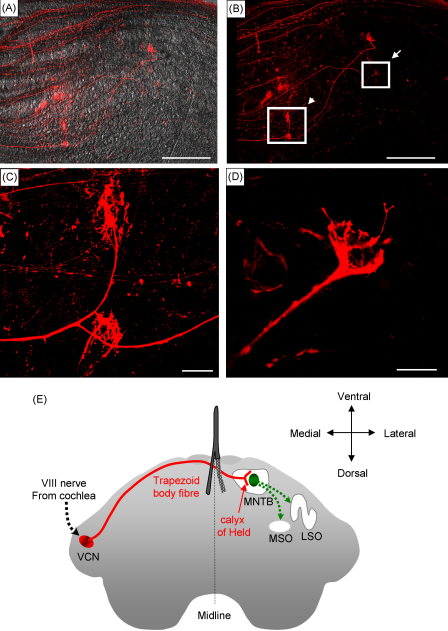
Neuronal tracing of dextran amine dye in brainstem slices. (A) Overlaid transmitted light and fluorescent image of trapezoid body fibres. (B) Same field of view as in A showing a fluorescent labelling of dextran amine dye within the trapezoid body fibres, 4 h after local electroporation and dye ejection at the midline. (C) Magnified area indicated by the arrowhead in B showing trapezoid body fibres with two labelled Held calyces. (D) Magnified area indicated by the arrow in B showing a trapezoid body fibre terminating onto a calyx of Held. Scale bar is 200 μm for A and B and 20 μm for C and D. (E) Schematic diagram of the auditory brainstem. The output from the ventral cochlear nucleus (VCN) projects across the midline to the contralateral medial nucleus of the trapezoid body (MNTB), giving rise to the calyx of Held presynaptic terminals. Principle neurones of the MNTB send inhibitory projections to the ipsilateral medial superior olive (MSO) and lateral superior olive (LSO) in the plane of the slice. The forked stimulating electrode is placed on the midline with one prong above and one below the slice.

**Fig. 9:**
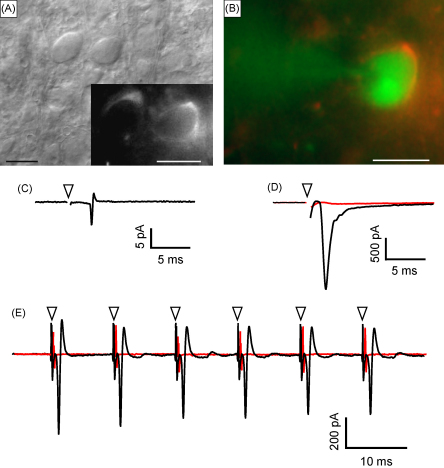
Combined ejection and local electroporation allows detection of functional synaptic connections in the medial nucleus of the trapezoid body. (A) differential interference contrast image of two postsynaptic cells of the medial nucleus of trapezoid body fibre. The inset shows their respective calyces labelled with fluorescent dextran amine previously delivered within the trapezoid body fibres using the double-barrelled electrode. Scale bars for both A and inset are 20 μm. (B) A postsynaptic cell of the medial nucleus of trapezoid body fibre is filled with lucifer yellow via the patch electrode (green) whereas the surrounding calyx is labelled with dextran amine (red). Scale bar is 20 μm. (C) Cell-attached recording of the labelled cell in B showing an action potential following single electrical stimulation of the trapezoid body fibres. (D) Same cell as in B and C recorded in whole-cell condition showing an excitatory postsynaptic current following a single electrical stimulation of the trapezoid body fibres at the midline before (black trace) and after (red trace) perfusing with 20 μM NBQX. Arrowheads in C, D and E indicate the location of stimulation artefacts that have been removed for clarity. (E) Cell-attached recording of a labelled neurone of the medial nucleus of the trapezoid body showing action potentials after stimulating the trapezoid body fibres at 100 Hz (black trace). The red trace shows the block of the action potentials after perfusing with 20 μM NBQX. All recordings were performed at a holding potential of −70 mV. (For interpretation of the references to colour in this figure legend, the reader is referred to the web version of the article.)

We used the combined ejection and local electroporation method to test whether introducing dextran amine locally within trapezoid body fibres allowed tracing presynaptic axons to calyx of Held terminals located about 700 μm away from the site of ejection, in the medial nucleus of the trapezoid body (Fig. 8E). Fig. 8B–D shows presynaptic calyx of Held terminals labelled with dextran amine 2 h after dye delivery. To confirm that dextran amine loading was due to cell membrane electroporation rather than dye uptake by damaged axons, we injected the same dye solution directly in the trapezoid body fibres by pressure ejection through the same pipette. Three hours after the pressure ejection we did not observe any neuronal labelling. Therefore electroporation allows for dextran amine penetration (see also Nagayama et al., 2007). Fig. 9 further shows that it was possible to obtain successful electrophysiological recordings from postsynaptic principal neurones of the medial nucleus of the trapezoid body connected to the labelled calyces. Fig. 9A and B show calyces labelled with fluorescent dextran amine and Fig. 9B also shows a postsynaptic cell of the medial nucleus of trapezoid body filled with lucifer yellow via the patch electrode (green). Cell-attached extracellular recording of the postsynaptic cell was made to confirm that the neuron identified by its labelled calyx does receive a synaptic input (Fig. 9C). The postsynaptic action potential could be detected as a current approximately 2 ms after the stimulation artefact. The presence of this current confirmed that the postsynaptic neurone had an active synaptic current. Fig. 9E shows another cell-attached recording of a postsynaptic cell that was identified by its labelled calyx. Trains of stimuli were delivered at 100 Hz resulting in postsynaptic action potentials firing in a reliable manner, as described for this fast relay synapse (Taschenberger and von Gersdorff, 2000). When whole-cell current recording was accomplished, the large glutamatergic postsynaptic current was clearly visible following stimulation (Fig. 9D). This current was blocked by 20 μM NBQX in both cell-attached and whole-cell recording (Fig. 9E and D respectively) and was therefore mediated by the activation of glutamatergic AMPA receptors as classically reported (Billups et al., 2002).

## 4 Discussion

One main problem in biological research is the introduction of macromolecules into cells without compromising cellular function. Current models for delivering exogenous macromolecules within a tissue have limitations due to complexity and variability of both tissues and cellular architecture. One approach, namely electroporation, achieves transient pore formation within cell membranes as membranes are subjected to brief electrical fields. Existing electroporation techniques within a tissue rely mainly on the separation of the anode and the cathode for the passage of the electrical current, with cells being electroporated *en masse* between those electrodes. We developed a method allowing efficient and focal delivery of exogenous macromolecules in neuronal tissue using simultaneous pressure ejection and local electroporation. With the proposed method, very few manipulations are needed. The method is readily accessible and requires either standard equipment that can be found in an electrophysiological laboratory or can be easily purpose built using a valve pressure ejection system coupled to a constant voltage supply.

### 4.1 Combining ejection with local electroporation allows local dye delivery and improves cellular viability

We report a simple method combining ejection with local electroporation through a single double-barrelled micropipette for efficiently introducing macromolecules into cells in culture or in slices. Since the ejection site also acts as an electrode, the coordinates of pore formation spatially coincides with the area of ejection. We show that a combination of ejection and electroporation is necessary to efficiently introduce reagents of MW of 10,000 *in situ* or *in vitro* within a confined area of 100–200 μm diameters, probably due to the confined electric field. We also show that it was possible to transfect locally HEK293 cells with an efficiency of 50% and also transfect cerebellar and hippocampal slices within confined areas of around 350–500 μm. The slightly larger diameters obtained in those latter conditions could be due to cells still dividing and migrating and/or to small compression resulting from the coverslip. The voltage used to introduce dextran amine or propidium iodide dyes (30 V) produced little tissue damage when electroporation was applied locally compared to when electroporation was applied throughout the whole tissue. Local electroporation is therefore less damaging compared to global electroporation as it limits damage to the cellular matrix and avoids immersing the tissue in ice cold PBS to avoid heat damage from the electroporation (Yang et al., 2004).

### 4.2 Combined ejection and local electroporation allows detection of functional synaptic connections

The ability to stimulate synaptic inputs within a brain slice and record from postsynaptic neurones has widely increased our understanding of synaptic transmission. However the process of cutting brain slices unavoidably damages many longer axons, making it very difficult to identify functional synaptic connections in certain brain areas. Within the medial nucleus of the trapezoid body, less than 10% of the cells retain viable synaptic inputs following the slicing procedure (Billups et al., 2002). We showed that presynaptic axons can be easily traced using local ejection of dextran amine combined to local electroporation of the presynaptic pathway. This allowed electrophysiological recording from pre-selected postsynaptic cells that were innervated by functional synaptic connections. Combining pressure with local electroporation provides an alternative possibility to tracing functional synaptic connections using calcium indicators (Billups et al., 2002). In this study, brainstem slices were loaded with fura-2AM and stimulation of the synaptic inputs caused intracellular calcium concentration to rise in postsynaptic neurones with active synaptic connections. Supra-threshold postsynaptic responses were an absolute requirement for detection of functional synapses by fura-2AM. Combining ejection with local electroporation is a valuable tool to detect synapses independently of the threshold of the postsynaptic responses. A previous study used simultaneous loose cell-attached stimulation and recording of action potentials to screen presynaptic cells while stimulating them selectively (Barbour and Isope, 2000). Whereas this technique also offers the possibility to study synapses with sub-threshold postsynaptic responses, establishing such recordings of synaptically connected neurones is also very time consuming. By comparison, the method described here provides a quicker way of detecting axonal pathways leading to presynaptic terminals. Using a single pipette to eject and electroporate also guarantees that both ejection and electroporation are made in the same precise location and avoids introducing another pipette into the preparation. This is especially useful when postsynaptic cells are subsequently recorded in close vicinity to the site of ejection and avoids repetitive stimulations of the synaptic network that could interfere with synaptic plasticity. Recently, Nagayama et al. (2007) used a patch clamp electrode and a clip attached to the mouse tail to perform local electroporation and pressure-eject synthetic Ca^2+^ dyes in various brain regions *in vivo*. Although their study found no differences in the field potentials before and after local electroporation, suggesting that function of local circuits is not compromised, it is possible that the design of the device contributes to creating inhomogeneous electric fields. Furthermore, in their study, electroporation pulses were delivered between 2 and 10 min. Using a double-barrelled electrode to perform local electroporation for a shorter period (5–10 s) is likely to minimise the risk of seeing indirect effects through activation of neighbouring cells. It also allows the dissection of spatially related effects that cannot be distinguished by systemic pharmacological manipulations or global electroporation. Local ejection and electroporation through a double-barrelled electrode into a defined location in the CNS could be very useful for gene therapy where low toxicity and minimal tissue are required.
